# PPARδ Inhibits Hyperglycemia-Triggered Senescence of Retinal Pigment Epithelial Cells by Upregulating SIRT1

**DOI:** 10.3390/antiox11061207

**Published:** 2022-06-20

**Authors:** Eun Ji Lee, Jun Pil Won, Hyuk Gyoon Lee, Eunsu Kim, Jinwoo Hur, Won Jin Lee, Jung Seok Hwang, Han Geuk Seo

**Affiliations:** College of Sang-Huh Life Science, Konkuk University, 120 Neungdong-ro, Gwangjin-gu, Seoul 05029, Korea; dongja@konkuk.ac.kr (E.J.L.); wjp0505@konkuk.ac.kr (J.P.W.); krci11@konkuk.ac.kr (H.G.L.); gennao@konkuk.ac.kr (E.K.); wlsdn91@konkuk.ac.kr (J.H.); windfall@konkuk.ac.kr (W.J.L.); mathking83@konkuk.ac.kr (J.S.H.)

**Keywords:** high glucose, peroxisome proliferator-activated receptor δ, premature senescence, reactive oxygen species, retinal pigment epithelial cells, resveratrol, senescence-associated β-galactosidase staining, SIRT1

## Abstract

Emerging evidence shows that peroxisome proliferator-activated receptor delta (PPARδ) plays a pivotal role in cellular aging. However, its function in retinal disease processes such as hyperglycemia-associated diabetic retinopathy is unclear. Here, we demonstrate that PPARδ inhibits premature senescence of retinal pigment epithelial (RPE) cells induced by high glucose (HG) through SIRT1 upregulation. A specific ligand GW501516-activation of PPARδ suppressed premature senescence and production of reactive oxygen species induced by HG in ARPE-19 cells, a spontaneously arising human RPE cell line. These effects were accompanied by the regulation of the premature senescence-associated genes *p53*, *p21*, and *SMP-30*. Furthermore, GW501516-activated PPARδ almost completely abolished the effects of HG treatment on the formation of phosphorylated H2A histone family member X (γ-H2A.X) foci, a molecular marker of aging. These inhibitory effects of GW501516 were significantly reversed in ARPE-19 cells stably expressing small hairpin RNA targeting PPARδ. Notably, GW501516 significantly increased the mRNA and protein levels of SIRT1, indicating that GW501516-activated PPARδ exerted its beneficial effects through SIRT1. In addition, GW501516 restored HG-suppressed SIRT1 expression, corroborating the role of SIRT1 in the anti-senescence function of PPARδ. The effects of PPARδ on HG-induced premature senescence and the expression of the senescence-associated genes p53, p21, and SMP-30 were mimicked by the SIRT1 activator resveratrol, but blocked by the SIRT1 inhibitor sirtinol. Collectively, these results indicate that GW501516-activated PPARδ inhibits HG-triggered premature senescence of RPE cells by modulating SIRT1 signaling.

## 1. Introduction

Peroxisome proliferator-activated receptor delta (PPARδ) is a ligand-inducible transcription factor implicated in diverse physiological and pathological processes, including aging [1,2,3]. This nuclear receptor functions as a biological modulator and regulates the expression of target genes by binding to a PPARδ-response element (PPRE), which is located in the regulatory regions of target genes [1]. PPARδ is ubiquitously expressed in diverse cell lineages including retinal pigment epithelial (RPE) cells [4]. It was recently postulated that PPARδ, activated by its specific ligand GW501516, exerts anti-senescence effects by modulating expression of its target genes [3,5]. GW501516-activated PPARδ confers resistance of vascular smooth muscle cells (VSMCs) to angiotensin II (Ang II)-induced senescence through phosphatase and tensin homolog deleted on chromosome 10 (PTEN)-mediated modulation of PI3K/Akt signaling cascades [2]. Notably, transcriptional upregulation of SIRT1 and antioxidant genes by PPARδ also inhibits Ang II-induced premature senescence of both human endothelial cells and VSMCs [3,5]. PPARδ also contributes to the resistance of human keratinocytes to ultraviolet B-triggered senescence [6]. Based on its potential role in regulation of cellular senescence [2,3,5,6], the possible beneficial effects of PPARδ on aging processes of RPE cells implicated in hyperglycemia-associated retinal damage must be evaluated.

The RPE, a monolayer of pigmented cells, is located at the interface of the neural retina and choriocapillaris. It plays a pivotal role in the strict control of the crossing of molecules into and out of the retina by forming the outer blood-retinal barrier [7]. The generation of reactive oxygen species (ROS) was increased in the sustained hyperglycemia, thereby contributing to the progression of diabetic retinopathy including the functional defects in the RPE which lead to the chronic disease state characterized by vision loss [8]. Although the connection between hyperglycemia and diabetic retinopathy has not been fully elucidated, high glucose (HG) levels in diabetic patients are closely associated with oxidative stress [8,9]. Development of diabetic retinopathy was led by excess high ROS levels which linked to the abnormal cellular functions [10]. Oxidative stress is closely related to the cellular senescence, termed stress-induced premature senescence, in diverse cell lineages including RPE cells [11,12]. Excessive ROS generation is causally linked with hyperglycemia-mediated senescence of various cell types [13,14]. Consistently, glucagon-like peptide-1 receptor-mediated inhibition of ROS generation elicits cytoprotective effects on hyperglycemia-exposed RPE cells, indicating that modulation of ROS generation is a potential therapeutic approach to prevent or manage diabetic retinopathy [15].

A NAD^+^-dependent class III protein deacetylase SIRT1 is a mammalian orthologue of yeast sir2 (silent information regulator 2) which coordinates complex gene expression programs by deacetylating diverse proteins including histones [16]. It functions as a critical modulator of cellular processes including aging, apoptosis, metabolism, and the cell cycle by interacting with a variety of substrates such as histones, p53, and p21 [17,18,19]. Two studies reported that SIRT1 has beneficial effects on oxidative stress-induced premature senescence and damage of RPE cells [12,20]. GW501516-activated PPARδ also increases SIRT1 promoter activity in human hepatocyte-derived cells and coronary artery endothelial cells [3,21].

Although numerous factors are associated with the execution of PPARδ actions, the effector molecules regulated by PPARδ in RPE cells are uncertain. We investigated PPARδ-responsive molecules associated with premature senescence induced by hyperglycemia in ARPE-19 cells, a cell line derived from the normal eyes of a 19-year-old male. We found that GW501516-activated PPARδ suppresses hyperglycemia-triggered premature senescence of ARPE-19 cells by upregulating SIRT1, which has been implicated in the modulation of lifespan [16].

## 2. Materials and Methods

### 2.1. Materials

Chloramethyl-2′,7′-dichlorofluorescein diacetate (CM-H2DCF-DA, Cat#287810), resveratrol (Cat#554325), and WY-14643 (Cat#681725) were purchased from Calbiochem (La Jolla, CA, USA). GW501516 (Cat#ALX-420-032) was purchased from Enzo Life Sciences (Farmingdale, NY, USA) Rosiglitazone (5-[[4-(2-[methyl-2-pyridinylamino]ethoxy)phenyl]methyl]-2,4-thiazolidinedione, Cat#71740) was provided by Cayman Chemical Company (Ann Arbor, MI, USA). An anti-β-actin (Cat#A2066) antibody, glucose (Cat#G8644), a senescence-associated β-galactosidase (SA β-gal, Cat#CS0030) staining kit, sirtinol (Cat#S7942), and lentiviral particles expressing non-targeting control (pLKO.1-puro Non-Target shRNA Control Transduction Particles, Cat#SHC001V) and PPARδ-targeting (TRCN0000350974, MISSION Lentiviral Transduction Particles) small hairpin RNA (shRNA) were obtained from Sigma-Aldrich (St. Louis, MO, USA). A monoclonal antibody specific for phosphorylated H2A histone family member X (γ-H2A.X, Cat#2577) and goat anti-rabbit IgG conjugated to Cy3 (indocarbocyanine, Cat#A10520) were purchased from Cell Signaling Technology (Danvers, MA, USA) and Invitrogen (Waltham, MA, USA), respectively. Monoclonal antibodies specific for p21 (Cat#SC-6246), p53 (Cat#SC-126), and SMP-30 (Cat#SC-130344), and a polyclonal antibody specific for SIRT1 (Cat#SC-19857) were purchased from Santa Cruz Biotechnology (Dallas, TX, USA).

### 2.2. Cell Culture

Human adult RPE ARPE-19 (American Type Culture Collection, Manassas, VA, USA) cells were routinely maintained in DMEM (5.6 mM glucose; WelGENE, Daegu, Korea, Cat#LM001-11) containing nutrient mixture F12 (Gibco, Carlsbad, CA, USA; one part DMEM, Cat#11330-032), 10 mM HEPES (Gibco, Cat#15630-080), 10% fetal bovine serum (Gibco, Cat#16000-044), and 1% antibiotics (100 U/mL penicillin and 100 μg/mL streptomycin, Gibco, Cat#15140-063) in a humidified atmosphere of 95% air and 5% CO_2_. Senescent RPE cell models using ARPE-19 cells were prepared by incubating in DMEM/F12 medium with 5.6 mM glucose (normal glucose, NG) until 80% confluent and maintained in serum-free medium for 24 h prior to switching to a medium containing 30 mM glucose (HG) for 48 h. In addition, for sufficient action of the PPARs ligand, WY-14643 (PPARα ligand, 10 μM), GW501516 (PPARδ ligand, 100 nM), or rosiglitazone (PPARγ ligand, 10 μM) was pre-treated 24 h before NG or HG treatment.

### 2.3. SA β-Gal Staining

Senescent cells were detected using a SA β-gal staining kit according to the manufacturer’s instructions. Briefly, ARPE-19 cells were treated with or without the indicated reagents for the indicated durations in 60 mm culture dishes. Following washing with phosphate-buffered saline (PBS), cells were fixed in a fixation solution (2% formaldehyde, 0.2% glutaraldehyde, 7 mM Na_2_HPO_4_, 1.47 mM KH_2_PO_4_, 137 mM NaCl, and 2.68 mM KCl) for 10 min at room temperature. Cells were then washed twice with ice-cold PBS and stained with SA β-gal staining solution (1 mg/mL X-Gal, 40 mM sodium phosphate, 40 mM citric acid, 5 mM potassium ferricyanide, 2 mM magnesium chloride, 5 mM potassium ferrocyanide, and 150 mM sodium chloride, pH 6.0) at 37 °C. Following incubation overnight, senescent cells were visualized under a fluorescence microscope (Olympus JP/1X71, Tokyo, Japan) equipped with a digital camera and scored. The number of stained cells was counted in six randomly chosen low-power fields per group.

### 2.4. Western Blot Analysis

Protein expression was evaluated according to a previously described method [22]. Briefly, ARPE-19 cells were exposed to the indicated reagents, washed with ice-cold PBS, and lysed using PRO-PREP Protein Extraction Solution (iNtRON Biotechnology, Seoul, Korea, Cat#17081). An aliquot of the cell lysate was size-fractionated by electrophoresis and transferred to immobilon-P polyvinylidene difluoride membranes (Merck, Darmstadt, Germany, Cat#IPVH00010). Following washing with Tris-buffered saline (TBS) containing 0.1% Tween-20, the membranes were blocked in TBS containing 5% non-fat skim milk and 0.1% Tween-20 at 37 °C for 1 h. Thereafter, the membranes were immersed in TBS containing each specific primary antibody and 0.1% Tween-20 and incubated overnight at 4 °C. The membranes were then reacted with a horseradish peroxidase-conjugated secondary antibody diluted to 1:7500 for 2 h at ambient temperature. After thorough washing with TBS containing 0.1% Tween-20, bands were visualized using WesternBright (Advansta Inc., Menlo Park, CA, USA, Cat#K-12045-D50).

### 2.5. Confocal Immunofluorescence Microscopy

To detect γ-H2A.X foci, ARPE-19 cells were seeded into 35-mm coverglass bottom dishes (SPL Life Science, Pocheon-si, Gyeonggi-do, Korea) as described previously [2]. Cells pretreated with GW501516 or a vehicle (DMSO) for 24 h were exposed to NG or HG. After incubation for 48 h, cells were fixed with 4% paraformaldehyde and permeabilized with PBS containing 0.1% Tween-20 for 3 min. Cells were then washed thrice with PBS, blocked with PBS containing 2% bovine serum albumin for 2 h at ambient temperature, and reacted with a primary antibody specific for γ-H2A.X overnight at 4 °C. Thereafter, secondary goat anti-rabbit IgG conjugated to Cy3 was applied for 2 h at room temperature. Following washing with PBS, cells were incubated in propidium iodide solution to stain nuclei. Finally, the coverglass was washed thrice with PBS and confocal imaging analysis was performed using an Olympus FV-1000 confocal fluorescence microscope.

### 2.6. Generation of ARPE-19 Cells Stably Expressing shRNA

Control and PPARδ-silenced ARPE-19 cells were generated by transduction of lentiviral particles expressing non-targeting and PPARδ-targeting shRNA as described previously [22]. Cells were seeded into 6-well plates and cultured for 20 h prior to transduction. Following transduction of lentiviral particles in the growth medium, transduced cells were selected by culture in the presence of 2 μg/mL puromycin for 8 days. Gene silencing was confirmed by immunoblot analysis.

### 2.7. ROS Measurement

The level of intracellular ROS was determined using the fluorescent probe CM-H2DCF-DA as described previously [23]. Briefly, cells were seeded into 35 mm coverglass bottom dishes (SPL Life Science) prior to treatment with the indicated reagents. Cells pretreated with GW501516 or DMSO for 24 h were exposed to NG or HG for 1 h and incubated with CM-H2DCF-DA for the final 30 min at 37 °C. Green fluorescence, which corresponded to the ROS level, was detected using an Olympus FV-1000 laser fluorescence microscope (Olympus, Tokyo, Japan) equipped with a 520 nm long-pass filter (Olympus, Tokyo, Japan).

### 2.8. RNA Extraction and Real-Time PCR Analysis

Total RNA was extracted from cells using TRIzol reagent (Invitrogen, Carlsbad, CA, USA, Cat#15596-018) and reverse-transcribed into cDNA using a TOPscript RT DryMIX Kit (Enzynomics, Seoul, Korea, Cat#RT201) to evaluate the levels of mRNA as described previously [24]. Briefly, real-time PCR was performed using equal amounts of cDNA in a Rotor Gene RG-3000 (Corbett Life Science, Sydney, Australia). The reaction was carried out in a volume of 20 μL and contained 10 pM primers and 2× Real-Time PCR Smart Mix (Solgent, Daejeon, Korea, Cat#SRH81-M40h). Following an initial denaturation for 10 min at 95 °C, the reactions were amplified by 45 cycles of 25 s at 95 °C, 45 s at 57 °C, and 35 s at 72 °C. The following primers were used: SIRT1 (NM_012238.5), 5′-TAGCCTTGTCAGATAAGGAAGGA-3′ and 5′-CTCGTACAGCTTCACAGTCAAC-3′; and GAPDH (NM_002046.7), 5′-CCTGCTTCACCACCTTCTTGAT-3′ and 5′-CATGGCCTTCCGTGTTCCTA-3′. The level of SIRT1 mRNA in each sample was normalized against that of GAPDH mRNA. The fold change in target gene expression relative to GAPDH expression was determined as described previously [25].

### 2.9. Statistical Analysis

Data are presented as means ± standard error (SE). Statistical significance between groups was assessed using one-way analysis of variance (ANOVA) followed by Tukey–Kramer tests. A *p*-value < 0.05 was considered to be statistically significant.

## 3. Results

### 3.1. Glucose Induces Premature Senescence of ARPE-19 Cells

**Figure 1:**
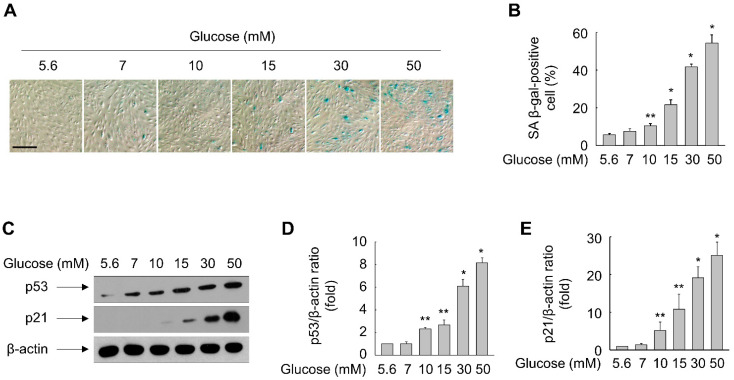
Glucose triggers senescence of ARPE-19 cells. (**A**–**E**) Cells grown in 60 mm dishes were exposed to increasing concentrations of glucose. Following incubation for 48 h, SA β-gal staining was performed (**A**) and SA β-gal-positive cells were quantified (**B**). The scale bar indicates 100 μm. Whole-cell lysates were subjected to immunoblot analyses (**C**) and the band intensities were quantified (**D**,**E**). Representative images from three independent experiments are shown. Quantitative data are presented as means ± S.E. (*n* = 3 for Western blotting and *n* = 6 for SA β-gal staining). * *p* < 0.01, ** *p* < 0.05 compared with the 5.6 mM glucose-treated group.

When ARPE-19 cells were exposed to glucose for 48 h, SA β-gal activity, which is a widely used biomarker of cellular senescence, was significantly enhanced in a concentration-dependent manner (Figure 1A,B). An increase in the SA β-gal-positive cells was significant at the concentration of 10 mM glucose and continued up to 50 mM glucose (Figure 1B).

To further characterize glucose-triggered cellular senescence, we measured the levels of p53 and p21, which are key proteins in the senescence pathway [26]. Consistent with the elevated percentage of SA β-gal-positive cells, glucose treatment increased the levels of p53 and p21 in ARPE-19 cells in a concentration-dependent manner (Figure 1C–E). These results suggest that glucose can damage the RPE by inducing cellular senescence.

### 3.2. Activation of PPARδ, but Not of PPARα or PPARγ, Suppresses HG-Triggered Premature Senescence of ARPE-19 Cells

**Figure 2:**
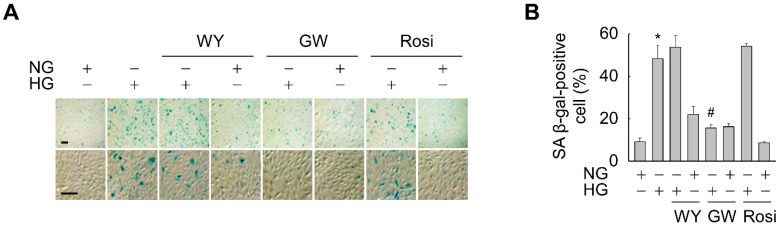
Ligand-activated PPARδ, but not PPARα or PPARγ, attenuates HG-triggered senescence of ARPE-19 cells. (**A**,**B**) Cells pretreated with WY-14643 (WY, 10 μM), GW501516 (GW, 100 nM), or rosiglitazone (Rosi, 10 μM) were exposed to NG (5.6 mM) or HG (30 mM). After incubation for 48 h, SA β-gal staining was performed (**A**) and SA β-gal-positive cells were quantified (**B**). Cells from at least six randomly chosen fields were scored (*n* = 100 per group). Scale bars indicate 100 μm. Quantitative data are expressed as means ± S.E. (*n* = 6). * *p* < 0.01 compared with the NG-treated group. # *p* < 0.01 compared with the HG-treated group.

The nuclear receptors PPARs have been implicated in the blockade of cellular aging [2,3,27]. Therefore, we evaluated whether activation of PPARs affects senescence of HG-treated ARPE-19 cells. SA-β-gal activity was significantly higher in HG-treated cells than in NG-treated cells. However, the HG-induced increase in SA β-gal-positive cells was significantly attenuated in the presence of GW501516 (PPARδ ligand), but not in the presence of WY-14643 (PPARα ligand) or rosiglitazone (PPARγ ligand), demonstrating that PPARδ is specifically involved in the suppression of HG-induced cellular senescence (Figure 2A,B).

**Figure 3:**
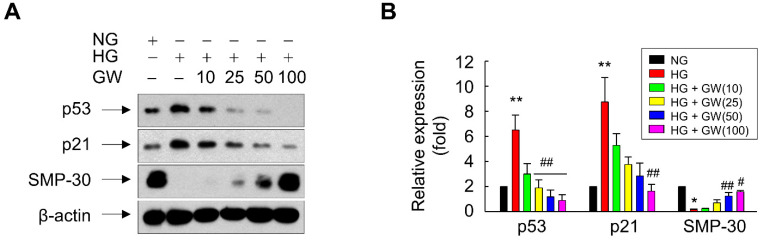
Ligand-activated PPARδ regulates expression of the senescence-associated marker proteins p53, p21, and SMP-30 in ARPE-19 cells. (**A**,**B**) Cells pretreated with a vehicle (DMSO) or 10–100 nM GW501516 (GW) for 24 h were exposed to NG (5.6 mM) or HG (30 mM). After incubation for 48 h, whole-cell lysates were prepared and subjected to Western blot analyses. Representative images from three independent experiments are shown (**A**) and quantitative data (**B**) are presented as means ± S.E. (*n* = 3). * *p* < 0.01, ** *p* < 0.05 compared with the NG-treated group. # *p* < 0.01, ## *p* < 0.05 compared with the HG-treated group.

**Figure 4:**
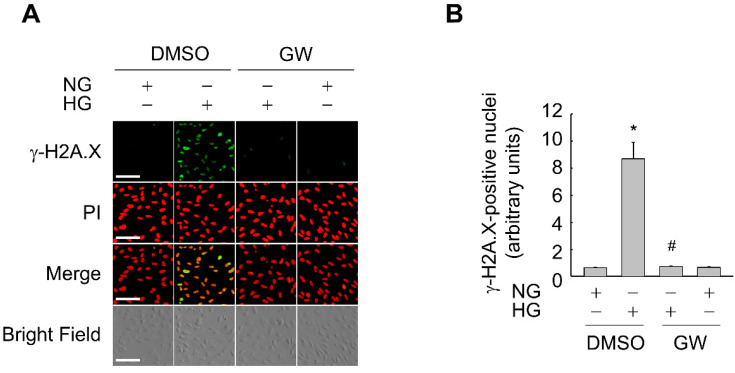
Ligand-activated PPARδ inhibits the HG-induced increase in the number of γ-H2A.X foci in ARPE-19 cells. (**A**,**B**) Cells pretreated with a vehicle (DMSO) or 100 nM GW501516 (GW) for 24 h were exposed to NG (5.6 mM) or HG (30 mM) for 48 h. Representative images from four independent analyses are shown (**A**). Scale bars indicate 100 μm. Cells were selected from at least four randomly chosen fields and γ-H2A.X-positive nuclei were scored (**B**). Data are expressed as means ± S.E. (*n* = 4). * *p* < 0.01 compared with the NG-treated group. # *p* < 0.01 compared with the HG-treated group.

In addition, GW501516 significantly attenuated the HG-induced upregulation of p53 and p21 in a concentration-dependent manner. By contrast, GW501516 significantly reversed the HG-triggered downregulation of SMP-30, another marker protein of cellular senescence [28], in ARPE-19 cells (Figure 3A,B). Consistently, GW501516 significantly attenuated the HG-induced increase in γ-H2A.X foci, another biomarker of cellular senescence [29] (Figure 4), corroborating the potential of PPARδ to inhibit HG-triggered cellular senescence.

**Figure 5:**
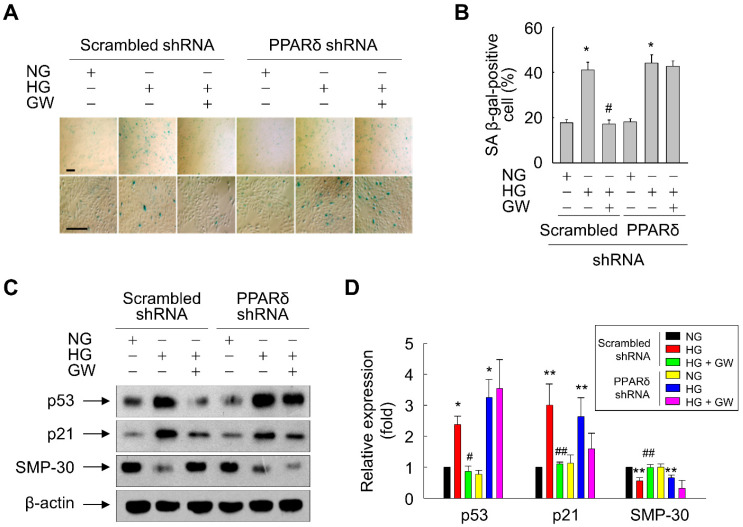
Transduction of PPARδ-targeting shRNA abolishes the effects of GW501516 on HG-induced senescence and expression of senescence-associated proteins in ARPE-19 cells. (**A**,**B**) Cells stably expressing scrambled or PPARδ-targeting shRNA were pretreated with 100 nM GW501516 (GW) for 24 h and then exposed to NG (5.6 mM) or HG (30 mM). After incubation for 48 h, SA β-gal staining was performed (**A**) and SA β-gal-positive cells were quantified in at least six fields (**B**). Scale bars indicate 100 μm. (**C**,**D**) Parallel immunoblotting for p53, p21, and SMP-30 (**C**) and densitometric quantification (**D**) were performed. Data are expressed as means ± S.E. (*n* = 3 for Western blotting and *n* = 6 for SA β-gal staining). * *p* < 0.01, ** *p* < 0.05 compared with the NG-treated group. # *p* < 0.01, ## *p* < 0.05 compared with the HG-treated group.

To further elucidate the anti-senescence function of PPARδ, the effect of GW501516 was assessed in ARPE-19 cells stably expressing PPARδ-targeting shRNA. The level of PPARδ in ARPE-19 cells was markedly reduced by transduction of PPARδ-targeting shRNA, but was unaffected by transduction of scrambled shRNA targeting nonspecific sequences (Supplementary Figure S1). As expected, the GW501516-mediated decrease in SA-β-gal-positive cells was almost completely reversed in cells stably expressing PPARδ-targeting shRNA, but not in cells stably expressing scrambled shRNA (Figure 5A,B). Furthermore, GW501516-mediated regulation of p53, p21, and SMP-30 levels in HG-exposed ARPE-19 cells was nearly abolished in cells stably expressing PPARδ-targeting shRNA (Figure 5C,D). These results indicate that GW501516 inhibits HG-triggered cellular senescence in a PPARδ-dependent manner.

### 3.3. GW501516-Activated PPARδ Attenuates HG-Triggered Generation of ROS

**Figure 6:**
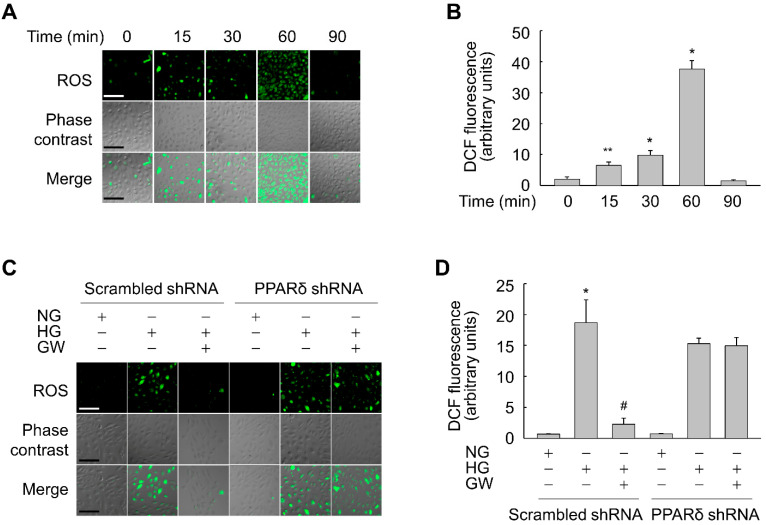
Ligand-activated PPARδ suppresses HG-triggered ROS generation in ARPE-19 cells. (**A**,**B**) Cells were incubated in the presence of 30 mM glucose for the indicated durations. (**C**,**D**) Cells stably expressing scrambled or PPARδ-targeting shRNA were pretreated with 100 nM GW501516 (GW) for 24 h and then exposed to NG (5.6 mM) or HG (30 mM) for 1 h. Following treatment with 10 μM CM-H2DCF-DA for the final 30 min, DCF fluorescence, which corresponded to the level of ROS, was detected by confocal laser fluorescence microscopy (**A**,**C**) and quantified (**B**,**D**). Scale bars indicate 100 μm. Representative images from three independent experiments are shown. The fluorescence intensity of DCF is expressed as means ± S.E. (*n* = 4). * *p* < 0.01, ** *p* < 0.05 compared with 0 min or the NG-treated group. # *p* < 0.01 compared with the HG-treated group.

ROS have been implicated in premature senescence [11]; therefore, we examined the effects of HG on ROS generation in ARPE-19 cells. When cells were exposed to HG, the level of intracellular ROS was significantly increased at 15 min, peaked at 60 min, and declined to the baseline at 90 min (Figure 6A,B).

To clarify the involvement of ROS in the anti-senescence action of PPARδ, we evaluated whether GW501516 affects the HG-induced ROS level. Exposure to HG significantly induced intracellular accumulation of ROS in ARPE-19 cells stably expressing scrambled shRNA. However, this HG-triggered increase in the ROS level was significantly suppressed in the presence of GW501516 (Figure 6C). Moreover, the GW501516-mediated reduction in the ROS level was significantly reversed in cells stably expressing PPARδ-targeting shRNA, indicating that GW501516-dependent inhibition of ROS accumulation is a primary factor in the anti-senescence action of PPARδ (Figure 6D).

### 3.4. GW501516-Activated PPARδ Increases mRNA and Protein Expression of SIRT1 in ARPE-19 Cells

**Figure 7:**
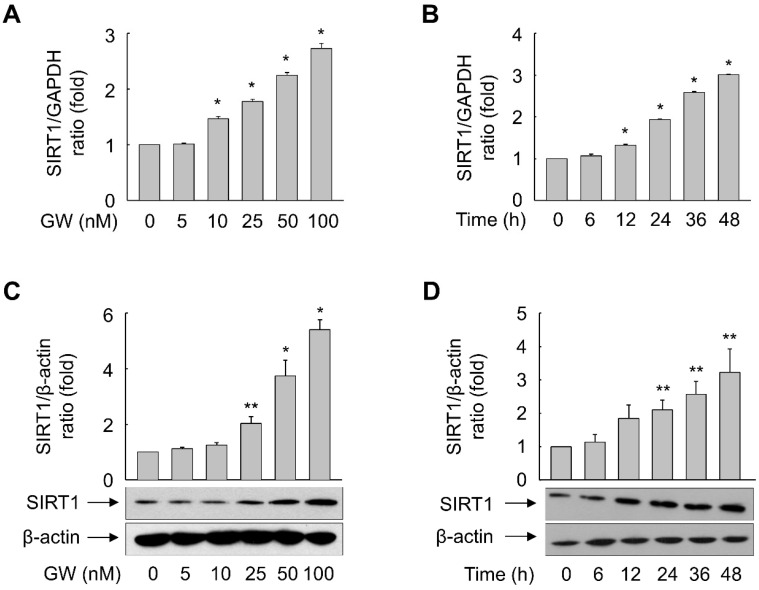
Ligand-activated PPARδ induces expression of SIRT1 in ARPE-19 cells. (**A**–**D**) Cells were incubated with various concentrations of GW501516 (GW) for 24 h (**A**,**C**) or exposed to 100 nM GW501516 (GW) for the indicated durations (**B**,**D**). Real time-PCR (**A**,**B**) and Western blot (**C**,**D**) analyses were performed. An image analyzer was used to quantify the band intensities, and the ratio of SIRT1 to β-actin was determined (**C**,**D**). Data are representative of three or four independent experiments and expressed as means ± S.E. * *p* < 0.01, ** *p* < 0.05 compared with 0 min or the untreated group.

Exposure of ARPE-19 cells to GW501516 increased mRNA and protein expression of SIRT1 in a time- and concentration-dependent manner. mRNA expression of SIRT1 was significantly increased after exposure to 10–100 nM GW501516 for 48 h (Figure 7A). When cells were treated with 100 nM GW501516, mRNA expression of SIRT1 was significantly increased at 12 h and peaked at 48 h (Figure 7B). Protein expression of SIRT1 was significantly increased upon treatment with 25–100 nM GW501516 for 48 h and peaked at 48 h upon incubation with 100 nM GW501516 (Figure 7C,D).

**Figure 8:**
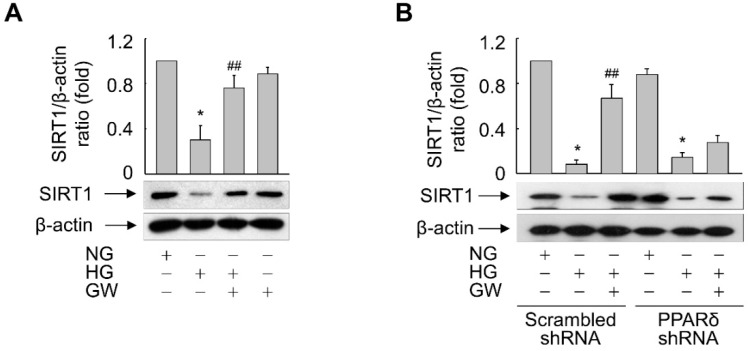
Ligand-activated PPARδ reverses the HG-triggered suppression of SIRT1 expression in ARPE-19 cells. (**A**) Cells pretreated with 100 nM GW501516 (GW) for 24 h were exposed to NG (5.6 mM) or HG (30 mM). (**B**) Cells stably expressing scrambled or PPARδ-targeting shRNA were pretreated with 100 nM GW501516 (GW) for 24 h and then exposed to NG or HG. After incubation for 48 h, whole-cell lysates were prepared and subjected to Western blot analyses. An image analyzer was used to quantify the band intensities, and the ratio of SIRT1 to β-actin was determined. Representative blots from three independent experiments are shown and quantitative data are shown as means ± S.E. (*n* = 3). * *p* < 0.01 compared with the NG-treated group. ## *p* < 0.05 compared with the HG-treated group.

To further elucidate the role of PPARδ in expression of SIRT1 upon HG treatment, the effect of GW501516 was assessed in ARPE-19 cells exposed to HG. HG treatment reduced the SIRT1 level and this effect was significantly reversed in the presence of GW501516 (Figure 8A). Furthermore, the GW501516-mediated recovery of SIRT1 expression was almost abolished in cells stably expressing PPARδ-targeting shRNA, indicating that SIRT1 expression is regulated in a PPARδ-dependent manner (Figure 8B).

### 3.5. SIRT1 Is Essential for PPARδ-Mediated Suppression of HG-Triggered Premature Senescence of ARPE-19 Cells

**Figure 9:**
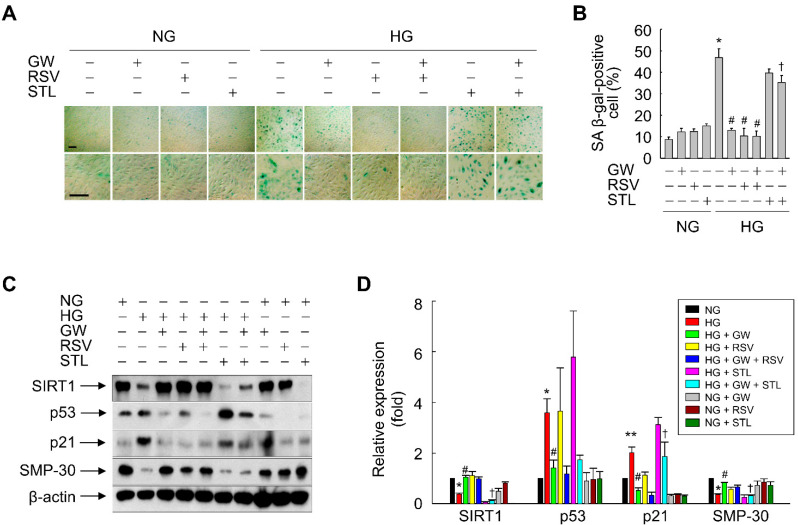
SIRT1 mediates the effect of PPARδ on HG-triggered senescence of ARPE-19 cells. (**A**,**B**) Cells pretreated with (+) or without (−) resveratrol (RSV) or sirtinol (STL) for 30 min were incubated in the presence of 100 nM GW501516 (GW) or a vehicle (DMSO). After incubation for 24 h, cells were exposed to NG (5.6 mM) or HG (30 mM) for 48 h and then senescent cells were detected by SA β-gal staining (**A**) and quantified (**B**). Scale bars indicate 100 μm. (**C**,**D**) Cells treated as described above were exposed to NG or HG for 48 h and then protein expression was analyzed by Western blotting (**C**) and quantified (**D**). Data are expressed as means ± S.E. (*n* = 3 for Western blotting and *n* = 6 for SA β-gal staining). * *p* < 0.01, ** *p* < 0.05 compared with the NG-treated group. # *p* < 0.01 compared with the HG-treated group. † *p* < 0.01 compared with the GW-treated group.

Expression of SIRT1 was reduced in cells exposed to HG and this effect was reversed in the presence of GW501516; therefore, we examined the functional significance of SIRT1 upregulation by PPARδ in inhibition of HG-triggered cellular senescence. The impact of SIRT1 activity on SA β-gal activity and expression of senescence-associated marker proteins in HG-exposed ARPE-19 cells was investigated. Inhibition of SIRT1 activity by sirtinol significantly attenuated the GW501516-mediated reductions in SA β-gal-positive cells and expression of p53 and p21 (Figure 9). By contrast, activation of SIRT1 by resveratrol significantly inhibited the HG-induced increases in SA β-gal-positive cells and expression of p53 and p21, similar to GW501516 (Figure 9). In addition, resveratrol reversed the HG-induced decreases in SIRT1 and SMP-30 expression to the same extent as GW501516. However, sirtinol did not counteract the effect of GW501516 on HG-exposed ARPE-19 cells, indicating that the SIRT1 pathway mediates the anti-senescence activity of PPARδ (Figure 9C,D). These results suggest that PPARδ inhibits HG-triggered premature senescence by increasing expression of SIRT1.

## 4. Discussion

PPARδ is a nuclear receptor that regulates expression of its target genes by functioning as a transcription factor to modulate diverse biological processes [1]. Although PPARδ plays a role in the senescence of vascular cells and human keratinocytes [2,3,5,6], little is known about its action in RPE cells. The present study demonstrated that GW501516, a specific agonist of PPARδ, attenuated HG-induced increases in the percentage of senescent ARPE-19 cells and the level of ROS. In addition, SIRT1, an anti-senescence factor [30], was significantly upregulated in GW501516-exposed ARPE-19 cells. shRNA-mediated downregulation of PPARδ antagonized the effects of GW501516 on senescence, ROS production, and SIRT1 expression in HG-exposed ARPE-19 cells. Furthermore, resveratrol, an activator of SIRT1, mimicked the anti-senescence action of GW501516 in hyperglycemia-exposed ARPE-19 cells, indicating that PPARδ inhibits cellular senescence by upregulating SIRT1.

We demonstrated that the anti-senescence function of PPARδ is linked with its induction of the intracellular longevity-related factor SIRT1 in RPE cells. This is consistent with the previous finding that ligand-activated PPARδ attenuates Ang II-triggered premature senescence of human VSMCs by modulating PI3K/Akt/Rac1 signaling through PTEN [2]. Furthermore, PPARδ-mediated transcriptional upregulation of SIRT1 and antioxidant genes, such as those encoding superoxide dismutase, glutathione peroxidase, and heme oxygenase-1, confers resistance of vascular cells to Ang II-triggered premature senescence [3,5]. In addition, pioglitazone, an activator of PPARγ, attenuates Ang II-triggered premature senescence of endothelial progenitor cells by regulating expression of the Ang II type 1 receptor [31]. Contrary to these results, another report showed that ligand-activated PPARγ accelerates cellular senescence by inducing expression of p16, a cell cycle inhibitor that promotes the onset of replicative senescence in human diploid fibroblasts [32]. Although the function of members of the nuclear receptor PPAR family in cellular senescence is controversial, the present study clearly demonstrates that activation of PPARδ by a specific ligand attenuates hyperglycemia-triggered premature senescence of RPE cells.

In diabetic retinopathy, excessive ROS generation is causally linked with hyperglycemia-mediated cellular senescence [20]. NADPH oxidase, but not mitochondria, has been implicated in ROS generation in hyperglycemia-exposed retinal capillary pericytes [33]. Therefore, production of ROS by NADPH oxidase seems to play a pivotal role in hyperglycemia-triggered senescence of ARPE-19 cells. Consistently, the present study showed that activation of PPARδ by the specific ligand GW501516 significantly attenuated hyperglycemia-triggered ROS generation in ARPE-19 cells.

The upregulation of SIRT1 by PPARδ is a critical event in the blockade of hyperglycemia-induced premature senescence of ARPE-19 cells. SIRT1, a longevity-related protein, is involved in multiple biological processes such as cellular senescence, apoptotic cell death, and energy metabolism [16,18,34,35]. Previous studies suggest that transcription of SIRT1 is regulated by complex mechanisms and calorie restriction is a primary regulator [36,37]. Induction of SIRT1 by PPARδ was originally demonstrated in human hepatocyte-derived cells [21]. That study reported that PPARδ regulates transcription of SIRT1 through specificity protein 1 (Sp1), but not through a PPRE. The functional significance of SIRT1 in cellular senescence has been demonstrated with GW501516-treated cells including vascular cells and keratinocytes [2,3,5,6]. The findings of the present study provide new insight into the primary role of PPARδ as an anti-senescence factor that inhibits hyperglycemia-triggered premature senescence of RPE cells.

## 5. Conclusions

Our observations suggest that activation of PPARδ by a specific ligand attenuates hyperglycemia-triggered premature senescence of RPE cells via upregulation of SIRT1, which reduces cellular ROS accumulation. Given that the expression of PPARδ target genes is involved in pathological changes [38], ligands that activate PPARδ might be key for controlling hyperglycemia-triggered cellular senescence.

## Data Availability

Data is contained within the article and supplementary material.

## Supplementary Materials

The following supporting information can be downloaded at: https://www.mdpi.com/article/10.3390/antiox11061207/s1, Figure S1: Effect of shRNA on expression of PPARδ. Cells were transduced with lentiviral particles expressing scrambled or PPARδ-targeting shRNA.
