# Supplementary material for: PPARδ Inhibits Hyperglycemia-Triggered Senescence of Retinal Pigment Epithelial Cells by Upregulating SIRT1
Source: Antioxidants (Basel). 2022 Jun 20;11(6):1207. doi: 10.3390/antiox11061207 (PMC9219651; doi:10.3390/antiox11061207)
Supplement: Supplementary file 1 [file antioxidants-11-01207-s001.zip › antioxidants-1744255-supplementary.pdf]

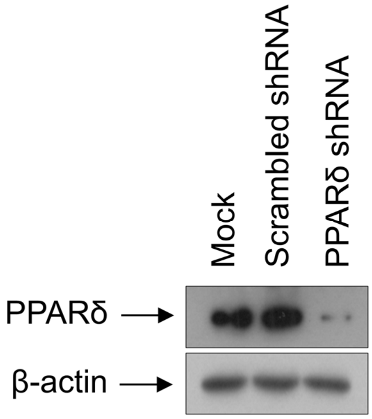

Supplementary Figure. S1. Effect of shRNA on expression of PPARδ. Cells were transduced with lentiviral particles expressing scrambled or PPARδ-targeting shRNA. Cells stably expressing shRNA were selected by culture in the presence of puromycin (2 μg/mL) for 8 days. Silencing of PPARδ was verified by Western blot analysis.
